# Nashville Correspondence

**Published:** 1871-01

**Authors:** 

**Affiliations:** Nashville


### OUR NASHVILLE CORRESPONDENT.

NASHVILLE, DEC. 15, 1870.

DR. T. S. POWELL:—*Dear Dr.* :—Very many will rejoice—

1st. That Atlanta is again to have a medical journal, and—

2d. That you are to conduct it, with our talented young friend, Dr. Goldsmith, as co-editor.

Receiving your prospectus to-day, I thought a line for your Journal, from this seat of medical learning, concerning medical men and medical things, might not be void of interest to your readers.

The University has a fine class, which gives active employment to a numerous corps professorial. Of the old men in the Faculty I need not speak, as they have been speaking for themselves a long time, and ought to be, and we believe are, satisfied with the public verdict. Those of them who left for a season have returned to their former colleagues, and the utmost harmony prevails. Of the "young faculty" T. B. Buchanan, Professor of Anatomy, is unquestionably the star—known wherever anatomy is cultivated as a genius of high order; he is moreover a splendid lecturer. Of commanding presence and felicitous elocution, he is the idol of every class. His elaborate productions as an anatomical artist, which so wonderfully enrich and distinguish the medical museum of the University, have made him famous, and given the student, as he beholds them, an exalted idea of the cunning hand and cultured brain, to which they owe their attractions. Prof. Madden is an earnest lecturer, with very pleasing manners and remarkable fluency. Prof. Nichol, of the last graduating class of the old *regime*, under the management of the elder Lindsley, a graduate in medicine from the University of Pennsylvania, for five years Assistant Surgeon in the United States Navy, and during the struggle of the South for independence, occupying a high position with the medical staff of her armies, brings to the discharge of the duties of his chair rare qualifications, upon which he draws without stint or mercy for material to enrich and illustrate his lectures. Prof. Callender was long the colleague of

Prof. Madden, in the Shelby Medical College, where he also greatly distinguished himself. A graduate of the Literary Department of the University, afterward long a student of Harvard, Massachusetts, a graduate from the Medical Department of the University of Pennsylvania, and for many years a Professor in Shelby Medical College, he came to his present Professorship while a young man, a distinguished veteran. He is among the most eloquent and impressive lecturers in the United States. He is also Superintendent of the Tennessee Hospital for the Insane. Prof. Briggs, though as Demonstrator, connected with the College from the beginning, as Professor, belongs to the young faculty. After the death of Prof. Buchanan, Prof. Briggs was transferred from the dissecting rooms to the Professorship of Physiology, where he greatly distinguished himself. After the death of Prof. Watson, he was transferred from Physiology to Obstetrics, and rose at once to the very head of his department, being, without doubt, inferior to no gynacologist of his country, as demonstrated by triumphant success throughout the domain of of woman's surgery. When the Professor of Surgery (Prof. P. F. Eve,) accepted the Professorship of Surgery in the Medical College of Missouri, Prof. Briggs was transferred to the Chair of Surgery, where he has acquired a distinction never before achieved by any man in so short a time. Prof. V. S. Lindsley, the youngest of the "young faculty," seven years ago was a student of medicine. He belongs to a family of students and scholars. His rapid elevation to fame and fortune, is, perhaps, without a parallel in American medicine. Thoughtful, graceful and profound, of easy elocution, he is destined to leave his foot-prints in the loftiest walks of his profession.

Five of the old Faculty—Bowling, Eve, Lindsley, Winston and Jennings; and five of the new—Briggs, Madden, Callender Nichol and V. S. Lindsley—ten in all—with one of the finest museums in the United States, splendid cabinets, and a good hospital, with magnificent college buildings, all free from tax, with a proud prestige, its future cannot be doubtful.

STUDENT.

---

REMEDY FOR DANDRUFF.—The *L' Union Medicale* gives the following formula for the treatment of dandruff - ℞—Nitric Acid 12 minims; Distilled water 3 ounces—mix and apply once a day.

